# Slovenian midwifery professionalization: Perception of midwives and related health professions

**DOI:** 10.18332/ejm/137664

**Published:** 2021-07-20

**Authors:** Polona A. Mivšek, Vanora Hundley, Edwin van Teijlingen, Majda Pahor, Valentina Hlebec

**Affiliations:** 1 Midwifery Department, Faculty of Health Sciences, University of Ljubljana, Ljubljana, Slovenia; 2 Centre for Midwifery, Maternal and Perinatal Health, Department of Midwifery and Health Sciences, Faculty of Health and Social Sciences, University of Bournemouth, Bournemouth, United Kingdom; 3 Faculty of Health and Social Sciences, Bournemouth University, Bournemouth, United Kingdom; 4 Faculty of Health Sciences, University of Ljubljana, Ljubljana, Slovenia; 5 Centre for Welfare Studies, Faculty of Social Sciences, University of Ljubljana, Ljubljana, Slovenia

**Keywords:** midwives, occupations, professionalism, professional boundaries, sociology of health

## Abstract

**INTRODUCTION:**

This article presents research into the professionalization of midwifery in Slovenia. Since recognition by related occupations is important for professions, this comparative study asked doctors and nurses in Slovenia about their perceptions of the status of midwifery.

**METHODS:**

A questionnaire survey was conducted with 300 Slovenian midwives, 666 nurses and 416 obstetricians. The questionnaire included statements covering traditional sociological notions of the profession (ethics, theory, power), and three notions based on new elements of professionalism (reflective practice, interdisciplinary working, and partnership with clients).

**RESULTS:**

Findings suggest that nurses perceived themselves to be less autonomous than midwives, and this partly explains why most nurses thought that midwifery should be a specialized course of study, after the general nursing diploma. Obstetricians claimed to support midwives, however, they did not give midwives credit for basic midwifery competencies and did not feel midwifery to be equal to their profession. Midwives revealed not to feel autonomous; they felt that nursing and obstetrics is jeopardizing independent midwifery practice.

**CONCLUSIONS:**

Slovenian midwifery was poorly evaluated in some attributes of professionalism, especially knowledge and autonomy. Even midwives themselves consider midwifery more occupation than profession. The autonomy of midwifery will be hard to achieve in the institutions of medical dominance. The study revealed that participants of all three groups are in a competitive relation and are poorly aware of the roles and competencies of the other two professions. Therefore, partially joined education might be beneficial in order to promote interprofessional collaboration in the future.

## INTRODUCTION

Over time all occupations strive to become professions as they are associated with high moral status, being respected, have the expertise, and social prestige^1^. While traditional professions represent the classical patriarchal values of power and self-sufficiency, ‘new’ professions are based on democratic professionalism^2^, promoting equality and partnership in the expert–patient relationship, reflective practice, and inter-professional collaboration. Instead of distance, the ‘new’ professionalism values cooperation where control is exchanged for support; knowledge is part of the therapeutic partnership^3^. The profession strives for cooperation with other independent professional groups, respecting each other, to achieve the best possible outcomes for patients^4^. In addition, the profession works in partnership with patients to ensure that care is appropriate and patient-centered.

The professionalization of an occupational group needs continuous political engagement^5^. No profession operates in isolation but instead is connected with others in the system of occupations. Relationships with similar professions play an important role in the professionalization of an occupational group^6^. Nursing and midwifery have achieved certain characteristics of a profession^7^. Pahor^3^ claims that the subordination of nursing and midwifery by medicine reflects the patriarchal subordination of women. At the same time, Witz^8^ suggests that it is the nature of the work (caring vs curing) that perpetuates this. Some claim that midwives will never achieve the professional status of medicine because they are within the same occupational hierarchy as medicine. It would require medicine to lose its status for midwifery (and other health occupations) to gain a more powerful position. Until then, midwifery is argued to be a para- or semi-profession^9,10^. In developing as a profession, medicine also defined the professional activity of midwives (defining normal and pathological) and taking over control of birth^11^. The final winning strategy was to shift the place of birth from home to the hospital^12^, resulting in most midwives working in an environment of medical dominance. Childbirth became medicalised^13^, as obstetricians claimed that pregnancy and birth could only be defined as uncomplicated only in retrospect^14^. The medical model treats birth as a potentially pathological process^15,16^.

In Slovenia, nurses have achieved certain elements of professionalization (self-regulation, autonomous education within universities and independent associations)^17^. Nursing started to develop a unique body of knowledge and its theories during the 1950s^18^, however, in some parts of Europe, including Slovenia, nurses remained under the supervision of medicine. The public often perceives them as doctors’ assistants, and their knowledge is regarded as simplified medical knowledge^3^. There are also tensions within the professional group; not all members want nursing to professionalize. Since they are not unified as a group, it is easier for medicine to subordinate them^19^. Our study focused on the opinion of nurses regarding the professionalism of midwives since, in Slovenia, their fields of work are not clearly defined. Historically^1,20^, nurses and obstetricians are competitors with midwives in pre-and post-natal care.

Today Slovenian midwives hold a similar position in the healthcare system to nurses, despite the fact these professions developed differently. Slovenian midwives, as in other parts of Europe, remained relatively autonomous until the 18th century^21^ when the rise of the medical profession began and reproductive health systems were established^22^. The battle between midwives and obstetricians is not only for control over birth. It is a battle over authoritative knowledge^23^, i.e. the authenticity of esoteric knowledge^24^ regarding pregnancy, birth and postpartum. ‘Ownership’ of childbirth, on the symbolic level, is connected with the jurisdiction and distribution of power^25^. By defining their scope of practice (pathology), obstetricians also demarcated that of midwifery (physiology)^26^. Although many women now give birth with a midwife in many countries, it is obstetricians that lead on guidelines and practices in hospitals^27^. As a result, women have also seen midwives as subordinate to obstetricians^15^. Since the maternity hospital is the territory of medicine, midwives became obstetric nurses - they adapted their titles, uniforms, role and even behaviour^28^. Nurses/midwives are authorized to do certain tasks due to the excessive workload of obstetricians, while obstetricians kept the cognitive jurisdiction (intellectual subordination)^6^. Midwives’ subordination is not only evident in their relationship with medicine but also nursing. In the new millennium, the academic cadre of midwifery teachers has grown stronger (achieving academic titles and taking over core midwifery subjects in the curriculum); however, these developments have not been long enough to establish midwifery’s research and theoretical basis. Few studies on the professionalization of midwifery have been conducted in Eastern Europe^29,30^, where midwifery developed differently from other high-income countries with more autonomous midwifery practice^27^.

Mivšek et al.^1^ had previously analyzed how midwives see their professional status, which is closely connected with the perception and recognition of related professional groups. We compared midwives’ views of Slovenian midwifery professionalization with clinical colleagues (nurses and obstetricians) who work with them. The research question was: ‘How do nurses and obstetricians perceive Slovenian midwives, particularly regarding midwives’ professional status?’.

## METHODS

### Study design

A postal questionnaire survey addressed six professionalization elements (autonomy, ethics, knowledge, reflective practice, interprofessional collaboration and partnership with patients) among midwives, nurses and obstetricians who work with midwives.

### Research instrument

The questionnaire comprised demographic questions and 4-point Likert scales were used to explore the six elements of professionalism. The questionnaire was developed using validated questions from foreign studies^31,32^ and the Slovenian tool was pilot tested before the main survey. The views from the midwives have previously been published^1^; this paper compares them with the perceptions of related health professionals with whom they work.

### Data collection

We approached all 300 midwives listed on the national nursing and midwifery register at the study time. This convenience sample represented 45.9% of Slovenian midwives by World Health Organization estimates^33^. Two different versions of the questionnaire were sent (midwives/ nurses) to nurses and midwives. Participants could choose which questionnaire to return. We wanted to assess if midwives felt affiliated with the midwifery profession or not. Devotion to a profession is one of the main characteristics of professionalism.

The register did not include information about the clinical areas in which members worked. Therefore, nurses were approached through all 14 Slovenian maternity hospitals and the Nursing Department in the only faculty that trains both professions. Since the study was self-financed, we were not able to include nurses from all Slovenian community centers, therefore a systematic sample was selected - every third community center in the region was invited, but if they refused to participate or did not employ midwives, the next on the list was approached. The sample included 63% of all community centers, representing all ten Slovenian regions. In total, 259 questionnaires were sent to community centers, 407 questionnaires to maternity hospitals and 21 to nursing teachers at the Faculty of Health Sciences.

Since obstetricians work in a variety of settings, including the private sector, questionnaires were distributed via their national professional association (to all 416 members). In the information sheet, they were asked to only complete the questionnaire if they worked with midwives.

The inclusion criteria were: midwifery, nursing or obstetrical education; registration with a professional association; currently practising (not retired, student etc.); and working as a midwife or working with midwives.

### Statistical analysis

Data were processed using SPSS, version 19. Dichotomized data (% agreement/disagreement with statements) are presented according to the professionalization elements of ‘new’^3^ and ‘old’^3^ professionalism. The proportion of respondents in each of the three professional groups who agreed with the statements was tested using the chisquared test. This was chosen because the groups were independent. Averages of Likert scales were calculated in each group for every element of professionalism. In order to do this, negative statements were recoded (i.e. higher score = greater professionalism).

## RESULTS

Altogether 152 midwives (50.7%) responded. Nurses returned 335 questionnaires (48.8% response rate) and 29.1% of obstetricians responded (n=121). After excluding probationers and retirees and considering nurses and midwives professional self-identification, the final analysis contained 127 midwives, 350 nurses and 101 obstetricians (Table 1).

**Table 1:** Demographic data of participants

*Characteristics*	*Midwives*	*Nurses*	*Obstetricians*
	*n (%)*	*n (%)*	*n (%)*
**Gender**			
Male	2 (1.6)	5 (1.4)	43 (42.6)
Female	125 (98.4)	345 (98.6)	58 (57.4)
**Age** (years)			
20-40	64 (53.3)	139 (42.5)	24 (26.2)
41-60	56 (46.7)	188 (57.5)	51 (56.1)
≥61	0 (0)	0 (0)	16 (17.6)
**Education level**			
Secondary	52 (40.9)	152 (44.2)	0 (0)
Undergraduate^a^	73 (57.5)	185 (43.8)	0 (0)
Postgraduate	2 (1.6)	7 (2.1)	101 (100)
**Employment**			
Community center			
Gynaecology	13 (40.6)	12 (9.0)	16 (100)
Community service	16 (50.0)	95 (71.4)	0 (0)
Working in different locations	1 (3.1)	4 (3.0)	0 (0)
Other	1 (6.3)	22 (16.7)	0 (0)
**Maternity hospital**			
Infertility treatment	1 (1.2)	2 (1.1)	2 (2.9)
Gynaecology	4 (4.9)	20 (10.9)	5 (7.1)
Intensive care gynaecology unit	2 (2.4)	9 (4.9)	1 (1.4)
Risk pregnancy	2 (2.4)	4 (2.2)	1 (1.4)
Delivery room	44 (53.7)	11 (6.0)	12 (17.1)
Postpartum ward	0 (0)	23 (12.6)	1 (1.4)
Intensive care postpartum ward	3 (3.7)	13 (7.1)	0 (0)
Intensive care neonatal ward	6 (7.3)	27 (14.8)	0 (0)
Pediatric department	1 (1.2)	6 (3.3)	0 (0)
Working in different locations	16 (19.5)	35 (19.2)	48 (68.6)
Other	3 (3.6)	32 (17.3)	0 (0)
**Private practice**			
Gynaecology	2 (50.0)	0 (0)	9 (100)
Community service	1 (25.0)	0 (0)	0 (0)
Other	1 (25.0)	1 (100.0)	0 (0)
Educational institution	2 (100.0)	8 (100.0)	0 (0)
**Duration of employment** (years)			
<5	48 (38.7)	103 (31.1)	17 (17.5)
5–15	25 (20.1)	80 (15.2)	36 (37.1)
16–25	15 (12.1)	91 (27.2)	24 (24.8)
>25	36 (29.0)	58 (17.5)	20 (20.6)
**Working post**			
Nurse (secondary education)	4 (3.3)	136 (40.8)	0 (0)
Midwife (secondary education)	44 (36.3)	9 (2.7)	0 (0)
BSc nurse (3-year program)	14 (11.5)	156 (46.8)	0 (0)
BSc midwife (3-year program)	58 (47.5)	16 (4.8)	0 (0)
Health Education Nursing (4-year program)	0 (0)	1 (0.3)	0 (0)
Nurse specialist (specialization after BSc)	0 (0)	5 (1.5)	0 (0)
Doctor (with finished specialist exam)	0 (0)	0 (0)	102 (100)
Assistant teacher	2 (1.6)	2 (0.6)	0 (0)
Other including high school teacher	0 (0)	8 (2.4)	1 (1.0)
**Work position**			
Leading position	19 (15.4)	62 (18.0)	52 (51.0)

a This is the highest possible level of midwifery education in Slovenia. There is no MSc or PhD level. In order to achieve MSc or PhD degree, midwives need to study abroad or choose a different discipline. Nursing can be studied at MSc level in Slovenia.

Within the group of nurses and midwives, females prevailed (98%), obstetricians were more gender equal (57.4% females); obstetricians were older, reflecting their length of employment. All obstetricians had completed postgraduate education (compared to only 1.6% of midwives and 2.1% of nurses) while almost half of midwives and nurses had finished only secondary school. In Slovenia, midwifery education was on a secondary school level till 1982; in 1996 a BSc midwifery program within the University of Ljubljana started; in between there was no midwifery education.

With regard to management, 18% of nurses and 15% of midwives held managerial posts (leading a team/department), compared to 51% of obstetricians. Approximately half of the hospital midwives worked in delivery rooms; most nurses in maternity hospitals worked on the postpartum wards, while obstetricians worked in several locations. In the community centers, nurses collaborated with midwives within community healthcare and obstetricians within gynaecological dispensaries.

### Perceptions of midwifery status regarding ‘old’ professionalism

Just over half of the participants thought that education is important to practise midwifery, with significantly more nurses stating that midwives should have at least BSc level education (Table 2). Midwives and nurses were more likely than obstetricians to state that practical knowledge is more important than theoretical knowledge for practising midwifery, but this finding was not statistically significant.

**Table 2:** Elements of old professionalism

*Elements*	*Agreement with the statements*			
	*Midwives*	*Nurses*	*Obstetricians*	*Chi-squared*
	*n (%)*	*n (%)*	*n (%)*	*n (%)*
**Knowledge**				
All midwives should have at least BSc level of education	74 (58.7)	233 (66.8)	56 (54.9)	15.526 (0.017)*
Practical knowledge is more important for midwifery than theoretical	78 (62.9)	208 (60.1)	57 (57.6)	2.491 (0.869)
Midwives are obstetric nurses	-	226 (66.5)	50 (50.5)	596.076 (0.001)*
All midwives should be nurses first	72 (58.5)	291 (84.3)	72 (73.5)	53.551 (0.001)*
Midwifery is specific (different than nursing or obstetrics)	76 (61.3)	175 (50.7)	63 (63.0)	8.427 (0.208)
**Ethics**				
Midwifery is important for the society	123 (98.4)	327 (94.2)	101 (99.0)	36.803 (0.001)*
Midwives practice according to code of professional ethics	123 (100.0)	315 (91.8)	99 (98.0)	9.289 (0.158)
**Autonomy**				
Midwives are competent for management of physiological pregnancy, birth and puerperium	101 (82.8)	237 (70.7)	44 (44.9)	42.786 (0.001)*
I/other health professionals, often seek professional advice from midwives	89 (72.4)	288 (87.5)	87 (89.7)	62.790 (0.001)*
Midwives are autonomous at work	52 (42.3)	166 (80.6)	68 (66.7)	466.478 (0.001)
Midwives would be willing to accept more professional responsibilities	97 (77.6)	254 (74.7)	58 (57.4	17.433 (0.008)*
Midwifery association represents midwives well	40 (32.8)	266 (82.4)	60 (73.2)	115.270 (0.001)*
Midwives are aware of the importance to be a member of professional association	102 (82.9)	140 (44.7)	34 (45.3)	532.708 (0.001)*
Midwives are closely connected within the professional association	-	279 (86.4)	66 (83.5)	548.427 (0.001)*
Society appreciates midwifery	81 (65.9)	261 (81.1)	74 (79.6)	14.500 (0.025)*
Society values other health professions more (than midwifery)	49 (40.2)	94 (28.2)	26 (26.5)	9.101 (0.168)
Midwives are proud of their profession	124 (100.0)	294 (90.7)	88 (93.6)	132.228 (0.001)*

* Statistically significant difference (p<0.05).

The majority in all groups thought that midwives should be nurses first, but nurses were significantly more likely to state this (84% vs 53% of midwives). Nurses were also more likely to think of midwives as obstetric nurses (66.5% vs 50.5% of obstetricians). Just over half of the participants agreed that midwifery is a specific profession. This contradicts the general agreement of all three groups that midwives are proud of their profession (100% agreement of midwives, 90.7% of nurses, and 93.6% of obstetricians).

Significant differences existed in the professions’ views on autonomy. Midwives were the least optimistic regarding their autonomy, while nurses rated midwives’ autonomy the highest – 80.6% agreed that ‘midwives are autonomous at work’. Although most (82.8%) midwives felt ‘competent to manage physiological pregnancy, birth and puerperium’, less than half of the obstetricians (44.9%) agreed. Obstetricians were also less likely to think that midwives would accept more professional responsibilities (57% vs 75% nurses and 78% midwives). Nevertheless, most obstetricians admitted frequently seeking professional advice from midwives (89.7%).

Only 32.8% of midwives agreed that their association represents them well. In contrast, the other two groups expressed more confidence in the midwifery professional association. Nearly all participants agreed that midwives ‘practise according to the code of professional ethics’^34^ and that ‘midwifery is important for society’, but there was a slight but statistically significant difference in the latter with nurses being the least convinced group (94.2%). Despite this, only 65.9% of midwives felt that society valued midwifery, which contradicts their response to the statement ‘society needs midwives’ to which they agreed 98.4%.

### Perceptions of midwives’ professional status related to ‘new’ professionalism

Approximately three-quarters of participants agreed with the statement ‘midwives practise informed decision making’ - more midwives agreed (73.2%) compared to obstetricians (67.3%) (Table 3). One-quarter of nurses and 15.3% of obstetricians claimed that midwives do not empower and enable women to decide the care they receive. However, a significant proportion of midwives reported that they sometimes could not fulfil women’s wishes because they cannot practise independently (73.2%).

**Table 3:** Elements of new professionalism

*Elements*	*Agreement with the statements*			
	*Midwives*	*Nurses*	*Obstetricians*	*Chi-squared*
	*n (%)*	*n (%)*	*n (%)*	*χ^2^ (p)*
**Partnership with women**				
Midwives’ practice informed decision	90 (73.2)	232 (72.3)	66 (67.3)	34.770 (0.001)*
Women’s satisfaction is more important to midwives than hospital rules	89 (73.6)	150 (47.8)	22 (22.9)	67.334 (0.001)*
If there is a conflict between doctor and a woman, midwives advocate for the woman’s rights	61 (50.8)	134 (42.9)	14 (14.7)	44.012 (0.001)*
Midwives often cannot fulfil woman’s wishes, because we are not independent	90 (73.2)	233 (70.4)	49 (50.0)	19.279 (0.004)*
Midwives do not allow women to have the power to decide	-	86 (27.4)	15 (15.3)	619.819 (0.001)*
**Interprofessional collaboration**				
Nurses professionally respect midwives	105 (84.0%)	330 (96.2)	76 (78.4)	42.513 (0.001)*
Obstetricians professionally respect midwives	99 (82.5)	251 (75.1)	99 (99.0)	31.742 (0.001)*
Midwifery is equal to my profession	-	-	17 (34.0)	543.000 (0.001)*
Obstetrics jeopardize midwifery	59 (48.0)	91 (27.9)	-	20.143 (0.001)*
Midwifery jeopardizes obstetrics	-	-	1 (1.0)	600.000 (0.001)*
Nursing jeopardizes midwifery	32 (26.2)	-	14 (13.9)	480.000 (0.001)*
Midwifery jeopardizes nursing	-	47 (13.8)	-	473.000 (0.001)*
Where I work, team cooperates well	108 (90.0)	291 (87.1)	97 (98.0)	28.010 (0.001)*
**Reflective practice**				
Midwives are doing their job professionally	-	334 (98.2)	93 (98.9)	582.047 (0.001)*
Midwives regularly control the quality of their professional work	-	284 (86.1)	62 (69.7)	579.210 (0.001)*
Midwifery practice is not evaluated by midwives, but other health professionals	81 (66.4)	150 (48.4)	39 (44.3)	17.300 (0.008)*
In case of professional negligence, midwives do not take the necessary responsibility	-	51 (16.2)	51 (53.1)	637.461 (0.001)*
In case of professional mistakes midwives are not protected	118 (95.9)	275 (85.9)	58 (65.2)	54.359 (0.001)*

* Statistically significant difference (p<0.05).

Midwives (73.6%) were more likely than the other professions to claim that women’s satisfaction is more important than hospital rules. They were also significantly more likely to report that they would advocate for a woman’s rights if there is a conflict between an obstetrician and a woman (50.8% midwives compared with only 14.7% obstetricians).

The interprofessional relations became clearer when analyzing statements regarding professional collaboration. Almost all agreed that professional collaboration was good, however, the group that was most satisfied with the interprofessional relationships was the obstetricians (98.0%), followed by midwives (90.0%) and then nurses (87.1%). Further analysis of interprofessional cooperation suggests that this is a complex issue. Almost all nurses reported to professionally respect midwives (96%), in comparison to one-quarter of obstetricians. Midwives reported feeling that their profession is jeopardized by nursing (26.2%) and some obstetricians agreed (13.9%). However, more midwives felt threatened by obstetrics (48.0%), and this feeling was supported by one-quarter of nurses (27.9%). The other two professions did not feel so threatened by midwifery (1.0% obstetrics and 13.8% nursing); a third of nurses and more than half of the obstetricians disagreed with the statement that midwifery is equal to their profession.

Almost all nurses and obstetricians agreed that midwives are performing their job professionally (99% agreement). Still, only 69.7% of obstetricians agreed that ‘midwives regularly control the quality of their professional work’, compared to 86.1% of nurses. More than half of the obstetricians agreed that ‘midwives do not take the necessary responsibility’ in cases of professional negligence (53.1%), but only 16.2% of nurses agreed with this statement. Significantly more midwives agreed that ‘midwifery practice is not always evaluated by midwives themselves’ (66.4% vs 44.3% of obstetricians and 48.4% of nurses).

Table 4 summarizes the averages for six elements of professionalization for all three professions. Midwives agreed that ethics is their strong professional element, with the average of the statements on the Likert scale at 3.3. Other professional groups agreed (3.2). The weakest element identified by all three groups was specific professional knowledge. Overall, the average estimate of midwifery professionalism (where all statements were included) was 2.9.

**Table 4:** Averages of 4-point Likert scales of all statements regarding six elements of professionalization

*Elements*	*Profession*			
	*Midwives*	*Nurses*	*Obstetricians*	*All*
Knowledge	2.6	2.4	1.7	2.5
Ethics	3.3	3.2	3.2	3.3
Autonomy	3.0	2.9	2.9	3.0
Collaboration with users	2.7	2.5	2.3	2.5
Interprofessional collaboration	3.2	3.2	3.3	3.2
Reflective practice	2.7	2.9	2.8	2.8

## DISCUSSION

The overall estimation of Slovenian midwifery professionalism by all three groups was quite positive; however, different occupations placed different values on the various elements of professionalization.

Slovenian midwifery is most poorly evaluated in the attribute of professional knowledge. Professional knowledge should be specific, having undergone rationalization and evaluation by the profession to ‘master the way of knowing’ and support the qualities, skills and behaviors needed for practice. However, in the case of Slovenian midwives, midwifery knowledge is not considered the sole property of midwives. This is probably because nurses perform postpartum care, and obstetricians do antenatal examinations. Another important criterion is that the development of professional knowledge requires a certain academic level of education, and this has been a challenge for Slovenian midwives^1^. Half of the obstetricians and nurses did not see the need for midwives to have degreelevel education. It is obvious that they still considered midwifery to be more of a vocation since more than 50% of participants thought that practical skills were more important for midwives than theoretical knowledge. The literature indicates that professionalism is marked with highly specialized theoretical knowledge, while skills are connected to craft^12^. However, in our study, the midwives were not a homogenous group. They did not share the value of academic knowledge - the difference in participant educational level was obvious; the opinion of graduate midwives differed in comparison to the opinion of midwives with secondary school education^1^. The large number of midwives educated at the secondary level could explain the anti-academic stream^19^ revealed in our study.

The feeling of belonging to a profession is very important for professionalism. This study identified that Slovenian midwives are not a cohesive group. The majority did not see themselves as a specific professional group, and half of them noted that all midwives should be nurses first. Some midwives even responded to the study as nurses, probably because they did not identify themselves as midwives. After consideration, we included them in the professional group they selected. The presentation of midwives as specialized nurses might arise also from the status that midwifery has in Eastern Europe^27^. Many midwives in our survey occupied posts of nurses and their status in health institutions was very similar to that of nursing. Professional roles are not clearly defined^20^; nurses and midwives are often considered the same profession. Even though autonomy reached average 3.0 on the four-point Likert scale, other results reflect a strongly hierarchical structure within the nursingmidwifery-medicine triangle.

The various reasons for weak professional identification include education since the majority of midwives were educated by nurses and obstetricians (there were no midwifery teachers before 2005); the midwifery scope of practice has not been clearly defined and overlaps with nursing. Midwifery positions are not recognized in all institutions and midwives are often employed in nursing positions.

Obstetricians and nurses also did not perceive midwifery practice as unique and even categorized them as obstetric nurses. Midwifery education is now reasonably autonomous^27^, however, its practical component is still a weakness. This is because students cannot acquire the required knowledge and skills in clinical settings since other professionals perform tasks that are typically undertaken by midwives elsewhere (antenatal examinations are taught by obstetricians, postpartum visits by nurses). Some claim midwifery knowledge is not specific enough to be an entity on its own^26^ and define it as a less demanding obstetrics approach^35^. Since very few in our survey would have been trained solely as midwives (after 2008) and not as a subspeciality of nursing, the professional socialization of midwives who perceive themselves as midwives and not obstetric nurses will only have influenced a small number of midwifery graduates.

Slovenian midwives have the right to perform five (out of ten) examinations in healthy pregnant women by law but are rarely employed in antenatal care, even if EU directives clearly state that management of normal pregnancies is midwives’ competency. Since midwifery education in Slovenia was abolished in the 1980s, there was a lack of midwives and consequently, they are poorly represented in pre- and post-partum care^1^. Most midwives are employed in maternity hospitals; in some, they can practise relatively independently in physiological births, however, the responsibility for the outcomes of labor still rests with obstetricians. Therefore, all midwifery practice must be in concordance with the medical profession^6^. This might be a reason why obstetricians did not recognize midwives as competent autonomous professionals for normal pregnancy, birth and postpartum^36^ and they estimated that midwives did not want more professional responsibilities. At the same time, obstetricians often seek their advice and claim to professionally respect midwives. The reason for their underestimation of midwifery competencies could be that they are still formally responsible for the birth outcomes. Nurses estimated that midwives are autonomous to a larger extent than midwives themselves. This might be why they would like to identify with them and thought midwives should be nurses first.

The autonomy of midwifery has a large impact on interprofessional relations. Good cooperation is possible only among independent professional groups that respect each other and are in equal positions^4^. Obstetricians in the study appreciated midwives but did not consider them to be equal. Midwives felt threatened by both related professional groups, which is not good for interprofessional collaboration^37^. Boundaries of subordinated professions are even more often challenged than those of dominant ones^10^.

Midwives in maternity hospitals are often subordinated by medicine^38^ and/or nursing^28^. Some midwives therefore assimilated and accept the medicalized practices^39^. In our study, some did not even feel competent in managing normal pregnancies and deliveries. This indoctrination shows a decline of professional midwifery identity that is crucial for the formation and maintenance of inter-professional boundaries^40^. Also, other studies report autonomy to be the weak characteristic of midwifery professionalism^41^. The lack of autonomy perceived by midwives can affect their attitude towards patients, although they are aware of the need to practise women-centered care. Three-quarters of midwives agreed with the statement that their ability to fulfil women’s wishes is curtailed because of the lack of independence. Only empowered professionals can empower patients^26^.

A professional should collaborate with and be assertive toward other professional groups^37^. However, midwives in our study feel jeopardized by nursing and medicine. In Slovenian midwives, we can question their ability to be assertive and advocate for women. Three-quarters of midwives claimed that women’s satisfaction is their priority. However, the other occupations, especially obstetricians, agree less with this. They are also the most restrained in the agreement that midwives’ practise informed decision. Some Slovenian midwifery practices in maternity hospitals are still not evidence-based, and informed decisions could sometimes be in contrast with institutional doctrine, which represents an ethical dilemma to midwives.

The relationship between midwifery and obstetrics is controversial^7^; maternity care in complicated cases needs medical intervention; therefore, it depends on obstetrics but midwifery fights domination by obstetrics over its own field of physiological childbirth^42^. The situation does not enable midwifery jurisdiction over its practice^43^. Gabe et al.^44^ claim that since medicine has the authority to define pathological (and so also the normal), it also sets boundaries for midwifery. Subordinate position of midwives could affect also how women see them. It could be a reason for midwives’ perception of their low value in society. They think the public appreciates more other health professionals. However, their perception is different to the views of the other two professional groups.

Individual members of the profession create the picture of a profession in public, but above all, this image is created by the professional association that advocates for the professional interests in relation to the state^10^. The association promotes the profession in public^32^ and with this raises the reputation of its members in society^6^. It can be, therefore, problematic that the competitive group is representing midwives. Slovenian midwives and nurses have a joint national association, however, with many more nurses, midwives form a small minority, which is reflected when making the political decisions where the conflict of interest exists (postpartum or prenatal care, for example). The consulting body of the Ministry of Health is for nursing only.

It might be the feeling of poor social recognition or ambiguous obstetricians’ attitude regarding the professional abilities of midwives and unsolved agreement regarding the competencies and field of work with nursing, why midwives think their association does not represent them well. In contrast, obstetricians and nurses feel that midwifery professional association links members well.

The association should control the activities of professionals since they are specialized and nonmembers cannot evaluate them^45^. In Slovenia, this seems problematic as midwifery practice is being evaluated by other professional groups, as claimed by almost half of the participants of each of the three groups. Midwives and nurses also largely agreed upon the statement that ‘in case of professional mistakes, midwives are not protected’. Obstetricians see it very differently. Half of them thought that ‘midwives do not take the necessary responsibility in the case of professional mistakes’; as formally responsible for the birth outcomes (also normal ones), they might feel unjustifiably exposed. Autonomy is closely connected with responsibilities^28^. However, not all Slovenian midwives are willing to accept more of them.

We must not forget the element that was perceived as the strongest of all six characters of professionalism by all three groups of participants - ethics. Also, by other studies, ethics was identified as a strong factor of midwifery professionalism^46^.

### Limitations

One limitation of our study is that terminology in the questionnaire can be interpreted differently by different health professionals (e.g. informed based decision, autonomy, etc.), therefore a glossary would be beneficial. Secondly, is the lack of the women's perceptions, as the perception of society regarding an occupation is very important. Since professions are usually protecting cultural values and are working for the greater good, society respects them and grants them special privileges. In our study, obstetricians and nurses thought that the midwifery profession was appreciated by society, while midwives thought that society respected other health professions more. The jurisdiction of the professional field is confirmed by society^43^. Therefore, users of midwifery services have great power in the enforcement of midwifery as a profession^47,48^, and future studies should incorporate their views.

### Recommendations

The recommendations that can be applied are as follows: 1) midwifery education has to be conducted by midwifery teachers in order to develop strong affiliation towards midwifery profession and identification with professional philosophy (physiology of pregnancy, birth and postpartum, partnership with woman etc.); 2) midwifery associations need to be strong political actors to promote and negotiate the position of its members in clinical settings (systemization of midwifery posts, clear scope of practice and competencies) and at the national level with the Ministry of Health; and 3) midwives need to develop an alliance with women. Partnership with women, as practising women-centered midwifery care empowers clients. And empowered and satisfied clients can negotiate for autonomous midwifery.

## CONCLUSIONS

Slovenian midwifery was marginalized with the abolition of midwifery education and the resulting lack of midwives in the past. This could be a reason for the weak traditional characteristics of ‘old’ professionalism. However, elements of ‘new’ professionalism offer new opportunities for midwifery, especially the concept of working in partnership with women. With public trust in midwifery work and with more active engagement of the professional association, the image of midwifery could be promoted in the media, and midwifery could be more visible at the governmental level. As a result, midwives’ professional status among fellow professionals and the wider society would also change. Future studies should evaluate women's views of midwifery in Slovenia.

## Data Availability

The data supporting this research cannot be made available, because they are a part of doctoral study of the first author and are co-owned by the Faculty where the candidate graduated.
